# Comparison of RNA-Seq and Microarray in Transcriptome Profiling of Activated T Cells

**DOI:** 10.1371/journal.pone.0078644

**Published:** 2014-01-16

**Authors:** Shanrong Zhao, Wai-Ping Fung-Leung, Anton Bittner, Karen Ngo, Xuejun Liu

**Affiliations:** 1 Systems Pharmacology and Biomarkers, Janssen Research & Development, LLC, San Diego, California, United States of America; 2 Immunology, Janssen Research & Development, LLC, San Diego, California, United States of America; 3 C.R.E.A.Te Integrative Systems Biology, Janssen Research & Development, LLC, San Diego, California, United States of America; Queen's University Belfast, United Kingdom

## Abstract

To demonstrate the benefits of RNA-Seq over microarray in transcriptome profiling, both RNA-Seq and microarray analyses were performed on RNA samples from a human T cell activation experiment. In contrast to other reports, our analyses focused on the difference, rather than similarity, between RNA-Seq and microarray technologies in transcriptome profiling. A comparison of data sets derived from RNA-Seq and Affymetrix platforms using the same set of samples showed a high correlation between gene expression profiles generated by the two platforms. However, it also demonstrated that RNA-Seq was superior in detecting low abundance transcripts, differentiating biologically critical isoforms, and allowing the identification of genetic variants. RNA-Seq also demonstrated a broader dynamic range than microarray, which allowed for the detection of more differentially expressed genes with higher fold-change. Analysis of the two datasets also showed the benefit derived from avoidance of technical issues inherent to microarray probe performance such as cross-hybridization, non-specific hybridization and limited detection range of individual probes. Because RNA-Seq does not rely on a pre-designed complement sequence detection probe, it is devoid of issues associated with probe redundancy and annotation, which simplified interpretation of the data. Despite the superior benefits of RNA-Seq, microarrays are still the more common choice of researchers when conducting transcriptional profiling experiments. This is likely because RNA-Seq sequencing technology is new to most researchers, more expensive than microarray, data storage is more challenging and analysis is more complex. We expect that once these barriers are overcome, the RNA-Seq platform will become the predominant tool for transcriptome analysis.

## Introduction

Since the invention of DNA microarrays in the 1990s, it has been the technology of choice for large-scale studies of gene expression. The ability of these arrays to simultaneously interrogate tens of thousands of transcripts has led to important advances in tackling a wide range of biological problems, including the identification of genes that are differentially expressed between diseased and healthy tissues, new insights into developmental processes, pharmacogenomic responses, and the evolution of gene regulation in different species [1]–[4]. Currently, microarrays remain the most popular approach for transcript profiling and can be readily afforded by many laboratories. Nonetheless, array technology has several limitations. For example, background hybridization limits the accuracy of expression measurements, particularly for transcripts present in low abundance. Furthermore, probes differ considerably in their hybridization properties, and arrays are limited to interrogating only those genes for which probes are designed.

RNA-Seq is the direct sequencing of transcripts by high-throughput sequencing technologies. It has shown strong potential to become a replacement to microarrays for whole-genome transcriptome profiling [5]–[9]. RNA-Seq has considerable advantages for examining transcriptome fine structure such as the detection of novel transcripts, allele-specific expression and splice junctions. RNA-Seq does not depend on genome annotation for prior probe selection and avoids the related biases introduced during hybridization of microarrays. However, RNA-Seq poses novel algorithmic and logistical challenges for data analysis and storage. Despite the fact that many computational methods have been developed for alignment of reads, quantification of gene and/or transcripts, and identification of differentially expressed genes [10], there is great variability in the maturity of these available computational tools.

To date, several studies comparing RNA-Seq and hybridization-based arrays have been performed [11]–[15]. Marioni, et al. estimated technical variance associated with Illumina RNA-Seq sequencing and compared its ability to identify differentially expressed genes with existing array technologies [14]. They found that RNA-Seq data on the Illumina platform was highly reproducible, with relatively little technical variation. The differentially expressed genes identified from RNA-Seq overlapped well with those identified by microarray. Fu et al. designed a study in which they used protein expression measurements to evaluate the accuracy of microarrays and RNA-Seq for mRNA quantification [15]. In that study, they used gene expression levels measured by a third technology – shotgun mass spectroscopy – to assess the relative accuracy of the two transcriptome quantification approaches with respect to absolute transcript level measurements, and found that RNA-Seq provided better estimates of absolute transcript levels. Details on RNA-Seq technology and the challenges and benefits associated with its technology and application were reviewed elsewhere [16]–[20]. Many recent studies were performed to run RNA-Seq and microarray in parallel with a focus on the concordance between them [11]–[13]. Our study focused on the differences, rather than consistencies, between the technologies and further investigated the reasons for observed discrepancies.

## Methods

### Human CCR6^+^ CD4 memory T cell RNA preparation

**Figure 1:**
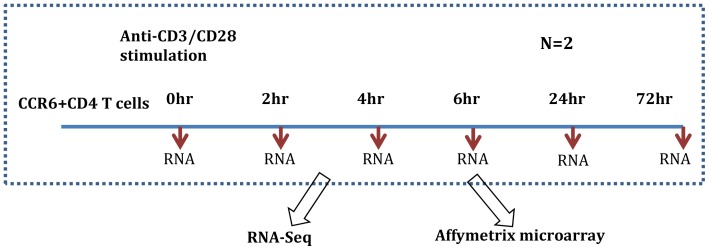
Experimental design. Human CCR6^+^ CD4 memory T cells were stimulated with anti-CD3/anti-CD28 coated beads under Th17 condition as described in Materials and Methods. RNA samples were prepared from cells collected at 0, 2, 4, 6, 24 and 72 hour post-stimulation. Gene expressions of these samples were studied with both Affymetrx microarray and RNA-Sequencing technologies.

Informed consent to participate in this study was obtained from the blood donor written permission using standard informed consent procedures and the use of human blood samples for research purpose was prior approved by Janssen R&D IRB (Institutional Review Board). Human PBMCs was purified from a healthy donor by step gradient centrifugation using Ficoll Pague (GE Healthcare Life Science). CD4^+^ memory T cells were purified from PBMCs through negative selection using the memory CD4^+^ T cell isolation kit (Miltenyi) followed by positive selection with anti-CCR6/biotin conjugates and anti-biotin magnetic beads (Miltenyi). Purified CCR6^+^ T cells were stimulated with anti-CD3 and anti-CD28 coated beads (Miltenyi) at 2∶1 bead/cell ratio in the presence of Th17 polarizing cytokines and antibodies including 10 ng/ml IL1β (R&D), 10 ng/ml IL23 (R&D), 30 ng/ml TGFβ1 (R&D), 10 µg/ml of anti-IL4 and anti-IFNγ (eBioscience). Stimulated T cells were cultured in 24 well tissue culture plate at 5×10^6^ cells/well in 1 ml of IMDM medium containing 10% serum replacement factor (Invitrogen) and supplemented with 2 mM glutamine, 1 mM sodium pyruvate, 10 mM HEPES, 1 mM MEM nonessential amino acid solution, and 100 U/ml each of penicillin G and streptomycin (Life Technologies). RNA was prepared from resting and stimulated T cells at different time points over a time course of 3 days. There were a total of six time points, with two biological replicates per time point (**Figure 1**).

### Microarray and RNA-Seq

Microarray-based transcriptome profiling was performed in the Janssen R&D microarray core facility using Affymetrix GeneChip HT HG-U133+ PM arrays. Gene expression was first measured at the probe set level (n = 54,715) using the RMA (Robust Multi-array Average) methodology on perfect match probes, followed by quantile normalization [21], [22]. Quality of the data was assessed using principal component analysis (PCA). Probe set annotation for the HT HG-U133+ PM array was downloaded from Affymetrix's website (see **Dataset S1**). 41,796 of the 54,714 probe sets were mapped to 20,741 genes, with 10,837 genes having more than one representative probe set. For each of these redundancies, the probe set with the greatest average expression across all samples was chosen to represent each gene.

RNA-Seq based transcriptome profiling was performed by Beijing Genomics Institute (Hong Kong), using the Illumina HiSeq™ 2000 platform. After extracting the total RNA from samples, mRNA was enriched by using the oligo (dT) magnetic beads, and was fragmented into short fragments (200∼500 bp) with the fragment buffer treatment. The first-strand cDNA was synthesized by random hexamer-primer with the mRNA fragments as templates. Buffer, dNTPs, RNase H and DNA polymerase I were used to synthesize the second strand. The double strand cDNAs, purified with QiaQuick PCR extraction kit, were used for end repair and base A addition. Finally, sequencing adaptors were ligated to the fragments. The fragments were purified by Agarose gel electrophoresis and PCR-amplified to produce the sequencing library. All reads were pair-end sequenced with an average insert size of 160 bp, and typical read-length of 90 bp. Primary sequencing reads produced by the Illumina HiSeq™ 2000 were next subjected to quality control. Data analysis was accomplished in two sequential tasks: (1) map all raw reads to the human reference genome hg19 using RefGene as the gene model, and (2) count the read fragments mapped to each individual gene and quantify expression by the corresponding RPKM (Reads Per Kilobase per Million mapped reads). The alignment algorithm was OmicSoft Sequence Aligner (OSA), a fast and accurate alignment tool for RNA-Seq [23].

In summary, RNA-Seq based transcriptome expression was measured as RPKM for 36,004 transcripts, representing 22,300 unique genes. The median RPKM in all 12 samples was 0.49, and 28.6% to 32.5% (average = 30.3%) of genes had RPKM value of 0 in each sample. In order to make the transcriptome profiling comparable between both platforms (RNA-Seq vs. Microarray), the RPKM values were floored at 0.047, followed by log2 transformation. After the transformation, the difference between the median expression and the floored (minimal) expression by RNA-Seq is equal to the difference between the median expression and the minimal expression by microarray.

### Differential expression profiling of transcriptome

Affymetrix probe design is based mainly upon the sequence clusters in the UniGene database [24], therefore the probe-sets don't cover all known genes in RefGene. In order to make a meaningful comparison, we only analyzed those genes common to both RefGene and the Affymetrix HT HG-U133+ PM array. As of October 2012, when our analysis was performed, there were 22,300 unique genes in the RefGene model, and 20,741 unique gene annotations for the Affymetrix HT HG-U133+ PM array. The number of genes common to both was 18,306 (see **Dataset S2**). In both platforms, differential expression of each common gene was first evaluated as an F-score generated by one-way ANOVA, with the underlying null (H_0_) hypothesis that the expression levels of the tested gene was identical among all six time points. Differential expression of each gene at any one of the five time points after Th17 activation was further determined as a log2 transformed ratio, using Dunnett's test, with the samples at 0 hour selected as the control group for all comparisons. All p values, associated with F scores or log2 ratios, were adjusted for multiplicity of testing by the Benjamini-Hochberg method [25].

The microarray expression data (see **Dataset S3**) after RMA normalization and the differential expression analysis results (see **Dataset S4**) are provided in supplementary materials. For RNA-Seq data, **Table S1** summarizes the metrics of read mapping for all 12 samples, and the corresponding read counts and RPKM are tabulated in **Dataset S5** and **Dataset S6**, respectively. The differential expression analysis result for RNA-Seq is provided in **Dataset S7**.

## Results

### Comparison of gene expression profiles between the two platforms

**Figure 2:**
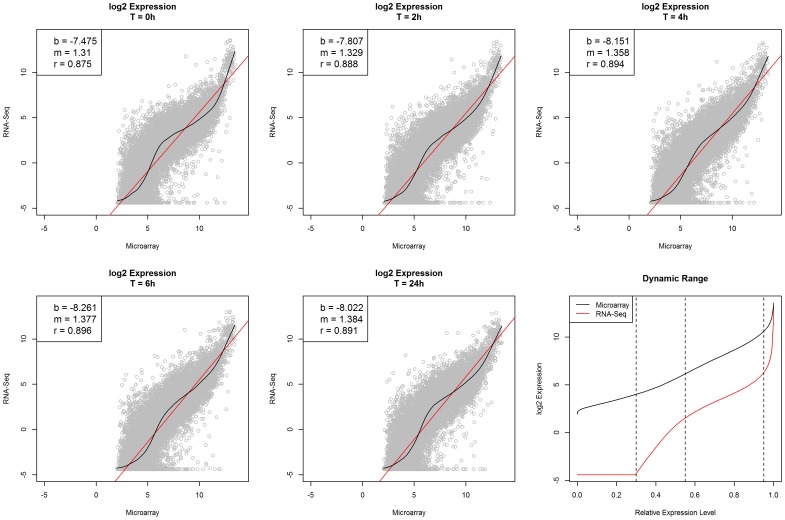
Comparison of expression profiles of 18,306 common genes between both platforms. Scatter plots show the averages (between biological duplicates) of log2 transformed expression values between two platforms, at each individual time point. The relationship between the expression profiles generated in both platforms is depicted as either a smoothing spline (black) or a linear regression line (red). The intercept (b) and the slope (m) of the linear regression line, and the correlation coefficient (r) are reported at the top-left corner in each plot corresponding to each time point. The plots show that the overall dynamic range of the 18,306 common genes generated by the two platforms is much broader in RNA-Seq (2.6×10^5^) than in microarray (3.6×10^3^). Similar dynamic ranges are displayed in both platforms for genes with relative expression level between 0.55 and 0.95. In each platform, the relative expression level of each gene was determined based on the average of log2 transformed expression values in all 12 samples.

The expression profiles of 18,306 common genes were compared between the two platforms, at individual time points (**Figure 2**). While high correlations (r = 0.88–0.90) were observed between the gene expression profiles generated by the two platforms at all six time points (similar results observed at T = 72 hour, data not shown), differences in expression profiles were also apparent between the two platforms, with a number of genes exhibiting relatively higher expression values in either platform.

The overall dynamic range was much broader in RNA-Seq (2.6×10^5^) than that in microarray (3.6×10^3^), especially at both the lower (with relative expression level less than 0.55) and the upper (with relative expression level greater than 0.95) ends. Note the relative expression level of each gene in the last plot in **Figure 2** was determined based on the average of log2 transformed expression values in all 12 samples. A relative expression level of 0.5 represents an underlying expression value in the middle of the range for all expression values. The vertical lines in the last plot of **Figure 2** indicate the relative expression levels at 0.30, 0.55 and 0.95 respectively. About 30% of expression values generated by the RNA-Seq platform were wither zero or below the floored level (0.047 RPKM). A broader dynamic range was observed in RNA-Seq compared to microarray at both ends, i.e. with relative expression level either less than 0.55 or greater than 0.95. A similar dynamic range was displayed in both platforms for genes with relative expression level between 0.55 and 0.95. Due to background hybridization or noise, all genes had an expression value in microarray, regardless of whether it was truly expressed or not.

The correlation coefficients between biological replicates range from 0.995 to 0.997 in microarray (see **Figure S1**), and the associated p-values with sample size of 18,306 genes are 0 (less than 1e-300). The corresponding correlation coefficients are 0.997 to 0.998 in RNA-Seq (**Figure S2**). Note that the correlation was calculated using log2 transformed expression values. For those genes with low expression levels, variability is higher in RNA-Seq. Clearly, RNA-Seq has a better correlation than microarray, as shown in **Figure S1** and **Figure S2**.

### Comparison of ANOVA results between the two platforms

**Figure 3:**
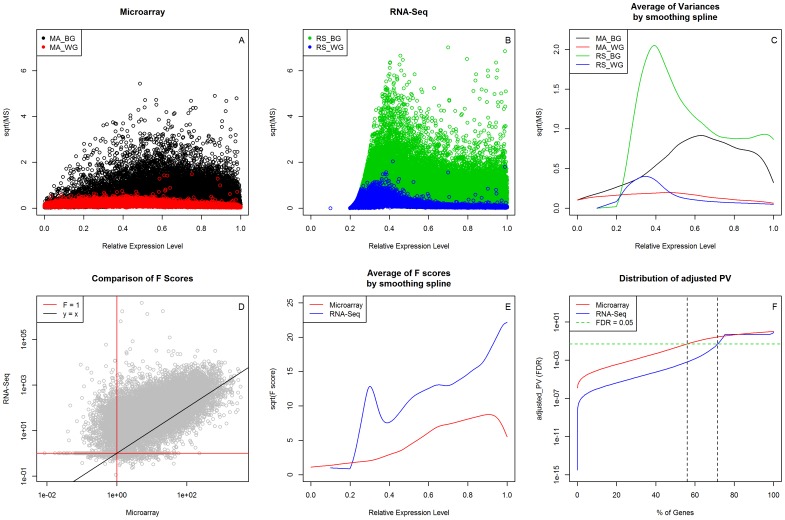
Comparison of ANOVA results between both platforms. Between-group (BG) and within-group (WG) variances are presented as the square root of the mean difference for each gene, and are plotted against the relative expression levels on both platforms, microarray (MA, panel A) and RNA-Seq (RS, panel B). The averages of variances (in square root, panel C) for between- and within-groups are plotted (panel C). The F-scores of 18,306 common genes are compared between two platforms (panel D), and the averages of F-scores (in square root, panel E) are also presented along with the relative expression levels by using smoothing spline for both platforms. The distributions of FDR-adjusted *p*-values, based on F-scores in both platforms are presented (panel F).

The variances, both between and within treatment group at all six time points, in log2 transformed expression values of the 18,306 common genes were analyzed by one-way ANOVA for both platforms (**Figure 3**). Within-group variances reflect data reproducibility, while between-group variances represent the sensitivity of platform to detect differential gene expression in response to T cell activation. For most genes, the between-group variances were larger than the within-group variances in both platforms (**Figure 3, A–C**), which is consistent with the expectation that many genes should be differentially expressed during the process of T cell activation. For genes with relatively low expression (relative expression level <0.47), within-group variances were higher in RNA-Seq than in microarray, representing lower reproducibility between the biological replicates. For genes with relatively high expression, within-group variances were lower in RNA-Seq, representing higher reproducibility (**Figure 3C**). The between-group variances exhibited similar patterns for genes with high expression in both platforms, whereas higher variances were observed in RNA-Seq for those genes with low expression (**Figure 3C**).

The capability to detect differential gene expression in both platforms was evaluated as an F-score generated by one-way ANOVA. Similar differential gene expression profiles were obtained in both platforms, as illustrated by high correlation coefficient (r = 0.718) between two sets of log-transformed F-scores (**Figure 3D**). 75.5% of genes exhibited higher F-scores in RNA-Seq, as compared to microarray. Positive correlations between F-scores and relative expression levels were observed in both platforms, indicating greater power in the detection of differential expression for genes with higher expression levels (**Figure 3E**). Using the F-score based, False Discovery Rate (FDR) adjusted P-value of 0.05, as a cut off, microarray and RNA-Seq selected 56.0% and 71.5% of genes, respectively, as differentially expressed among the six time points (**Figure 3F**).

### Comparison of differential gene expression profiles between the two platforms

Selection criteria for differential expression required genes to have fold-change greater than 2.0, FDR-adjusted *p* less than 0.05, and expression value greater than the median of values in all common genes (RPKM of 0.49 for RNA-Seq, and intensity of 40.2 for microarray) in at least one condition. **Table 1** summarized the differentially expressed genes at five time points after Th17 activation. Despite the high overlap between microarray and RNA-Seq results, there are also many differentially expressed genes that are unique to either platform. Our selection criterion for whether a gene is differentially expressed is very sensitive to a 2-fold change cut off. The overall similarity between the two platforms becomes more evident if we draw a heat map of fold change for those differentially expressed genes which are close to, but lower than, 2-fold.

**Table 1:** Comparison of differentially expressed genes identified from RNA-Seq and microarray.

Comparison	Gene Expression	RNA-Seq unique	Common	Microarray unique
2 hr vs 0 hr	Increased	911	602	801
4 hr vs 0 hr	Increased	1439	983	1325
6 hr vs 0 hr	Increased	1680	1135	1460
24 hr vs 0 hr	Increased	2290	1258	1480
72 hr vs 0 hr	Increased	2696	1441	1668
2 hr vs 0 hr	Decreased	2172	551	608
4 hr vs 0 hr	Decreased	3920	1545	1611
6 hr vs 0 hr	Decreased	3818	1597	1677
24 hr vs 0 hr	Decreased	2442	1282	1547
72 hr vs 0 hr	Decreased	2396	1374	1712

Note: Selection criteria for differential expression required genes to have fold-change greater than 2.0, FDR-adjusted p less than 0.05, and expression value greater than the median of values in all common genes (RPKM of 0.49 for RNA-Seq, and intensity of 40.2 for microarray) in at least one condition.

As displayed in **Table 1**, more genes were detected as differentially expressed in RNA-Seq as compared to microarray, especially for those that were down-regulated. The pattern of the number of differentially expressed gene across time points for RNA-Seq was interesting. It was noted that at late time points, i.e. 24 and 72 hours, the number of up-regulated genes increased, while the number of down-regulated genes decreased in RNA-Seq (compared to the expression changes at 4 or 6 hours). In contrast, in microarrays no apparent increase or decrease was observed if we compare the differentially expressed genes at 24 or 72 hours with those at 4 or 6 hours.

**Figure 4:**
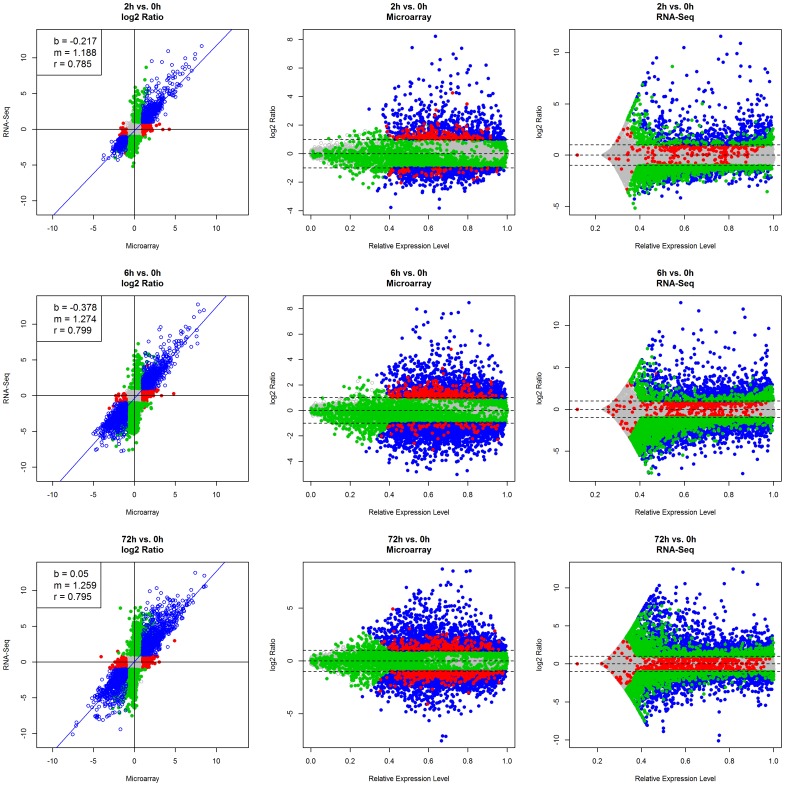
Comparing differential expression profiles of 18,306 common genes between microarray and RNA-Seq. Scatter plots of log2 transformed ratios (vs. baseline at T = 0 h) between both platforms at selected time points (T = 2, 6, and 72 hour) show similar results are observed at T = 4 and 24 hour. Genes that are specifically differentially expressed in microarray or RNA-Seq are colored in red and green respectively, and genes that are differentially expressed in both platforms are colored in blue.

Differential gene expression profiles of 18,306 common genes at each of the five time points following Th17 activation were compared between the two platforms (**Figure 4**, and data not shown). Similar differential gene expression profiles were obtained in both platforms at each time point, as illustrated by high correlation coefficient (r = 0.78–0.80) between the two sets of log-transformed ratios. However, the magnitude of differential expression was greater in RNA-Seq than in microarray, as indicated by the slopes (m = 1.18–1.27) at each time point. The distribution of differentially expressed genes, either platform specific or common to both platforms, was independent of their expression levels (**Figure 4**). Also, while a large number of genes were identified as differentially expressed in both platforms (colored in blue in **Figure 4**), there were still a number of genes specifically detected as differentially expressed in only one platform (colored in red and green, respectively, in **Figure 4**). There are several reasons for platform-dependent measurement of differentially expressed genes. First, as shown in **Figure 2**, the differences in expression profiles for some genes were apparent between the two platforms, and accordingly, different fold changes are calculated and reported. Second, for genes with very low or very high expression levels, RNA-Seq is more likely to detect the changes at two different conditions, as we will demonstrate later. Third, a microarray probe might hit some, but not all, isoforms of a gene, and as a result the reported fold change of the probe set does not necessarily represent the expression change of the entire gene. Probe set *205277_PM_at* is a case in point, which we will discuss in the Discussion section, as well as all of the inherit biases of microarray and RNA-Seq in detection of differential expression.

### Additional benefits achieved by RNA-Seq

**Figure 5:**
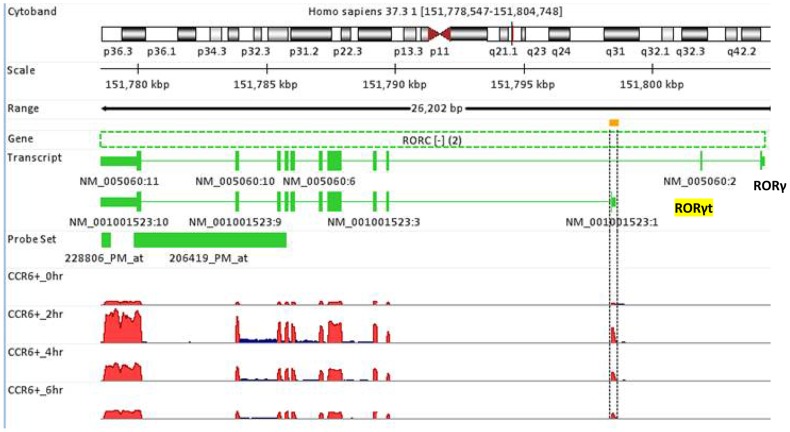
Detection of splicing variants with RNA-Seq approach. Human RORγand RORγτ are the two isoforms of the RORC nuclear receptor generated from alternative splicing of the gene. RNA-Seq results showed that RORγ and RORγτ are encoded in the minus strand of the gene and their mRNA transcripts share most of the exons except for one or two exons at the 5′ end. RORγ mRNA utilizes specific exons 1 and 2 whereas RORγt mRNA has its specific exon 1, and the two transcripts are driven by their distinct promoters. In microarray studies, these two isoforms were indistinguishable since the two probe sets *228806_PM_at* and *206419_PM_at* hybridize with the exon regions that are common for these two isoforms. In contrast, RNA-Seq showed the specific expression of RORγt but not RORγ in CCR6^+^ CD4 T cells.

Multiple transcripts generated from the same gene via alternative splicing is a common phenomenon in evolution and some of these variant transcripts have been shown provide differential functions which may have important implications to the survival and physiological response of the organisms. One of the key advantages of RNA-Seq is that it can differentiate the expression of individual isoforms in transcriptome profiling. RORγt is an orphan nuclear receptor playing an important regulatory role in promoting differentiation of CD4 T cells into pro-inflammatory T helper 17 (Th17) cells [26]. RORγt and RORγ are two isoforms derived from the same gene RORC by alternative splicing. In contrast to the functional role of RORγt in T cells, RORγ is involved in metabolism and expressed in other cell types such as adipocytes and hepatocytes. In the microarray platform, probe sets *228806_PM_at* and *206419_PM_at* hybridize to the RORC gene in regions that are common for both RORγt and RORγ transcripts and therefore cannot differentiate the expression of these isoforms. As shown in **Figure 5**, it is evident from RNA-Seq results that RORγt was the dominant isoform expressed in CCR6^+^ memory CD4 T cells.

**Figure 6:**
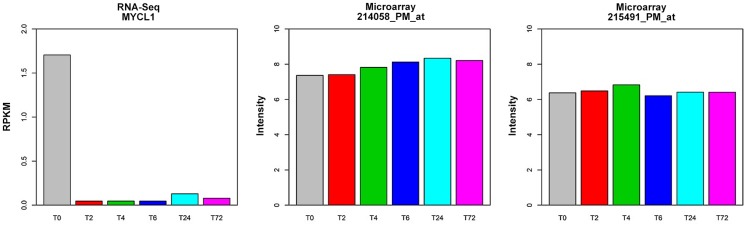
Comparison of RNA-Seq and Affymetrix microarray in detection of genes expressed at low levels. Expression of MYCL1 was at low levels in CD4 T cells and the subtle change in MYCL1 expression in the process of T cell activation cannot be detected by microarray approach (center and right panels). The high sensitivity of RNA-Seq approach allows detection of a more than 32-fold decrease in MYCL1 mRNA expression upon T cell activation (left panel).

**Figure 7:**
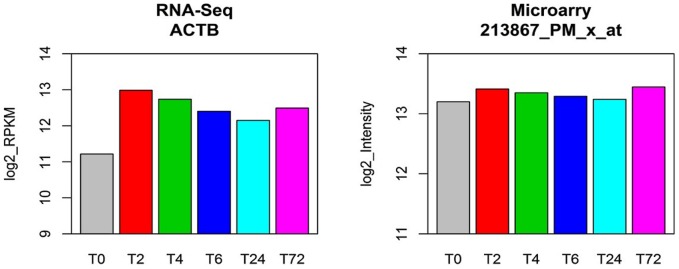
Comparison of RNA-Seq and Affymetrix microarray in detection of genes expressed at high levels. β-actin (ACTB) is expressed at high levels in all conditions as measured by both RNA-Seq and microarray. Microarray detected similar levels of ACTB between resting and activated T cells whereas RNA-Seq showed a 2 to 4-fold increase in activated T cells. ACTB has been reported to be 5.3-fold up-regulated in *in-vitro* stimulated lymphocytes measured by quantitative polymerase chain reactions [26].

Comparing to microarray platform, RNA-Seq seems to be more sensitive in direct measurement of low abundant transcripts (**Figure 2**) as well as in detection of changes in expression of these transcripts under different conditions (**Figure 3E**). An example of a low abundant transcript MYCL1 in T cells was demonstrated in **Figure 6**. RNA-Seq results showed that MYCL1 was expressed at low levels (with a RPKM of 1.7) prior to activation. Following T cell activation, its expression decreased further, with a level that was only 3% of the resting T cells at 2 hour after stimulation. Microarray failed to detect any changes in MYCL1 expression in T cell samples at all time points. On the other hand, microarrays are prone to “hybridization saturation” for highly abundant genes. Under this circumstance, microarray cannot give reliable quantitative measurements of subtle changes of high abundant genes (**Figure 3E**). As shown in Figure 7, ACTB was expressed at high levels in all conditions as measured by both RNA-Seq and microarray (**Figure 7**). β-actin (ACTB) is a highly conserved protein that is involved in cell motility, structure, and integrity, and has been used extensively for normalization of gene expression data. Microarray studies showed a stable level of ACTB expression among all samples tested, whereas RNA-Seq clearly demonstrated that ACTB expression was increased 2 to 4-fold in activated T cells when compared to resting cells at 0 hour. The fold-change in our RNA-Seq data was consistent with previous reports indicating that ACTB expression was 5.3-fold up-regulated in activated lymphocytes detected by quantitative polymerase chain reactions (qPCR) [27].

**Figure 8:**
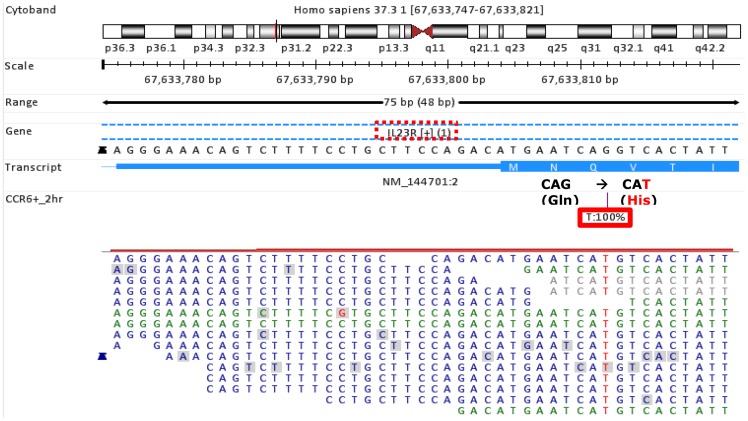
Detection of gene polymorphism with RNA-Seq approach. A single nucleotide change was identified in the IL23R gene of this donor from sequence reads in RNA-Seq. The change results in a Gln to His mutation at the third amino acid of the N-terminal of IL23 receptor.

In addition to differential expression studies, RNA-Seq is also capable of identifying single nucleotide variants (SNV) in human populations and genetic polymorphism have been shown to be important information in identification of defective genes associated with inherited diseases. Compared to array-based genotyping platforms, sequencing-based technology such as RNA-Seq have two key advantages in detecting genetic variants: (1) no prior knowledge on potential variants is required; and (2) detection is genome-wide even for rare SNPs. As shown in **Figure 8**, the donor was found to have a mutation in the IL23 receptor (IL23R) gene sequence, which resulted in a Gln to His change at the 3rd amino acid at the N-terminal of the receptor. The change corresponded to *rs1884444* in the dbSNP database. IL23R is expressed on a number of immune cell types including T cells and natural killer (NK) cells (http://ghr.nlm.nih.gov/gene/IL23R). When IL23R binds to its ligand IL23, a series of signalling events are triggered inside the cell influencing both innate and adaptive immune responses. It would be of interest to investigate the potential change in cellular response to IL23 in donors expressing this IL23R variant identified from our RNA-Seq data analysis. Our RNA-Seq data also confirmed that there existed a soluble form of IL23R in Th17 T cells in addition to the complete trans-membrane IL23R [12]. This new isoform is shorter, and truncated at exon 6. The sequencing depth is almost doubled from exons #1 to #6 compared to the rest of the exon regions, and this pattern can be easily understood when both isoforms are expressed. The soluble isoform IL23R has been further confirmed by RACE (rapid amplification of cDNA ends) [12].

### Probe set issues: redundancy, annotation and selective coverage

**Figure 9:**
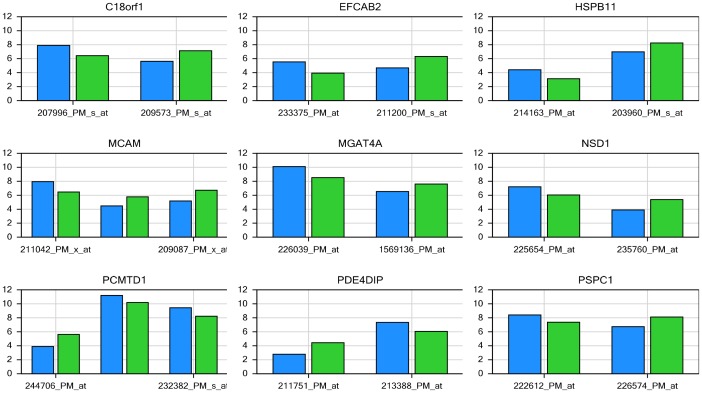
The controversy of redundant probe sets in microarray, and inconsistent results were obtained in Affymetrix microarray. The bars in blue represent genes expressed at 0-axis indicates gene expression levels in log_2_ scale.

The fact that multiple probe sets correspond to the same gene is both a blessing and a curse to data analysis. Usually, these redundant probe sets agree with each other but it is not uncommon when they do not yield a consensus, or even conflict with each other, as demonstrated in **Figure 9**. In this figure, the blue and green bars represent gene expression levels at 0 hour and 2 hour, respectively. Interpretation of whether the expression for those genes increased or decreased during the early stage of T cell activation is dependent upon the reporting probe sets. Since it is biologically impossible for a gene to increase and decrease simultaneously, at least one of the probe sets is necessarily inaccurate.

**Figure 10:**
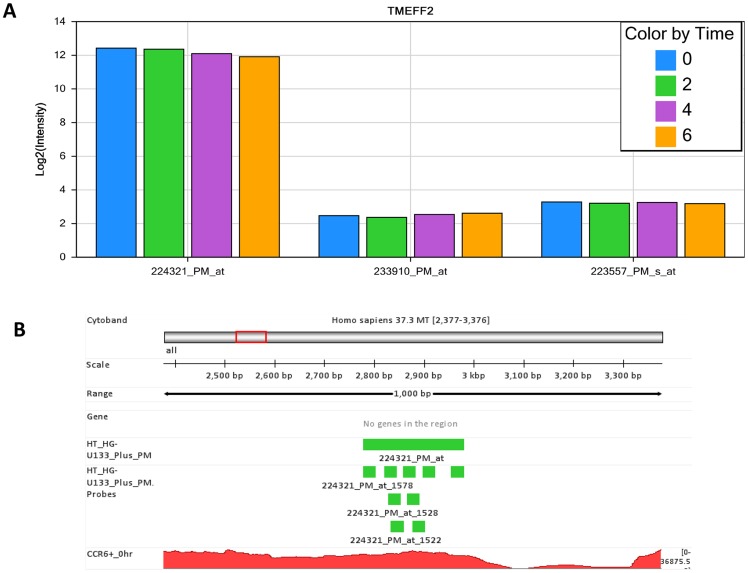
Inaccurate annotation of probe set 224321_PM_at resulted in conflicting results between Affymetrix microarray and RNA-Seq approaches for TMEFF2 expression. In Affymetrix microarray studies, probe set *224321_PM_at* showed high expression of TMEFF2 at 0, 2, 4 and 6 hours. However, the high expression reported by probe set *224321_PM_at* is supported neither by *233910_PM_at* nor by *223557_PM_s_at*. In contrast, there was no detectable expression of TMEFF2 in RNA-Seq studies. As a matter of fact, the probe set *224321_PM_at* is mapped to a genomic region that is unrelated to TMEFF2, and thus this Affymetrix probe set is inaccurately annotated.

**Figure 11:**
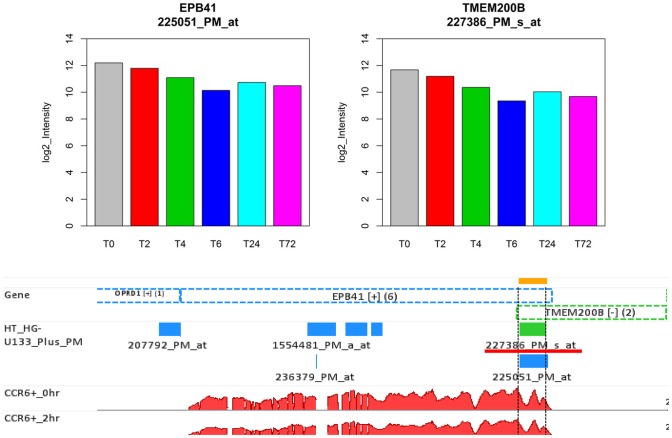
The gene expressions of EPB41 and TMEM200B are reported by microarray (top panel) and RNA-Seq (bottom panel). Probe set *227386_PM_s_at* targets the overlapping region of both gene EPB41 and TMEM200B (bottom panel). RNA-Seq clearly shows high expression of EPB41, but no expression of TMEM200B. The high expression of EPB41 is also shown by probe set *225051_PM_at* (top panel). Because the Affymetrix library file associates probe set *227386_PM_s_at* with only TMEM200B, it inaccurately reports high expression values for this gene.

In our microarray dataset, probe set *224321_PM_at* indicated that TMEFF2 was highly expressed at all time points (**Figure 10**). However, a contradictory result was found with RNA-Seq, which detected no expression for this gene. While investigating this discrepancy between the two platforms, we mapped *224321_PM_at* to human genome hg19. We found the probe set *224321_PM_at* more accurately falls in a genomic region different from TMEFF2, and concluded that the annotation for *224321_PM_at* is in error. In fact, the other two probe sets for TMFF2, *233910_PM_at* and *223557_PM_s_at*, measured only background signal, supporting the RNA-seq finding that there was no expression of TMFF2. Another example is the association between probe set *227386_PM_s_at* and TMEM200B, which more accurately targets the overlapping region of genes TMEM200B and EPB41 (**Figure 11**). Our RNA-Seq dataset very clearly showed high expression of EPB41, but no expression for TMEM200B. When using the Affymetrix annotation for *227386_PM_s_at*, we therefore incorrectly assigned expression of EPB41 to TMEM200B in our microarray dataset.

**Figure 12:**
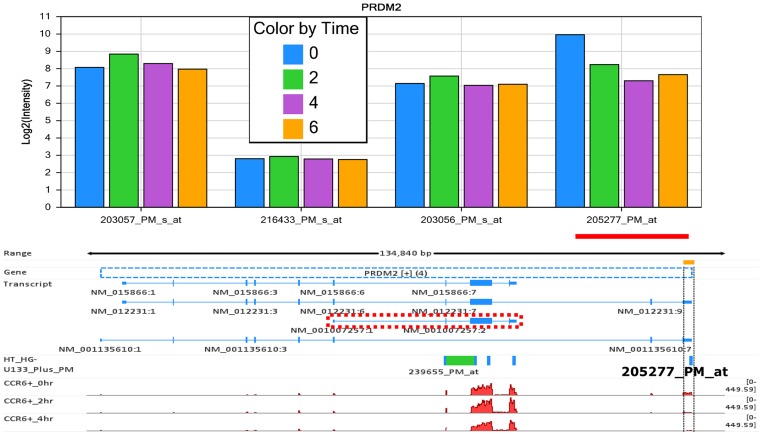
The 6.3-fold decrease from 0 hour to 4 hour reported by probe set *205277_PM_at* in microarray can only represent the change of two of the isoforms (NM_012231 and NM_001135610), but does not reflect the expression change for the entire PRDM2. On the other hand, the reported 6.3-fold decrease was not supported by peer probe sets, either.

Ideally, a probe set could target all alternatively spliced isoforms of the same gene. In practice, a probe set quite often targets only some, but not all, of the isoforms - or worse yet, might instead target an intron region of a gene. In such a situation, the reported change by this probe set does not truly reflect the entire gene. For instance, our microarray dataset detected a 5.7-fold increase for PDE6D between 0 hour and 2 hour, but RNA-Seq detected an insignificant decrease. The reason for this conflict is that the microarray probe set for PDE6D, *231065_PM_at*, actually targets an intron region. **Figure 12** illustrated another common scenario. For PRDM2, probe set *205277_PM_at* reported a 6.3-fold decrease from 0 hour to 4 hour. However, the decrease was not supported by other probe sets targeting PRDM2. There were 4 known isoforms for this gene, and transcript NM_001007157 was the most dominant isoform in terms of expression level according to read mapping in RNA-Seq data. Probe set *205277_PM_at* hit minor isoforms NM_012231 and NM_001135610, and thus, the reported 6.3-fold decrease represented only the change of the two minor isoforms, not the entire gene. In microarray data analysis, a probe set is usually assumed to correspond to a gene, and accordingly, the reported change is considered to reflect the gene expression change. This assumption is reasonable for the majority of probe sets, but not all of them.

## Discussion

A reasonable approximation of the values derived by microarray probe sets are the composite of three signals: 1) specific signal produced by the originally targeted labelled transcript, 2) cross-hybridization signal produced by transcripts that have non-perfect, but still significant, sequence similarity with the probe set, and 3) non-specific background signal, which is present in the absence of any significant sequence similarity. Because of background noise and cross-hybridization, microarrays have difficulty detecting genes with low expression level, and thus cannot distinguish “no” from “low” expression. Microarray probe intensity is assumed to be proportional to the concentration of the transcript, but also depends on the affinity of the probe under the given hybridization conditions. This affinity is determined to a large extent by the actual nucleotide sequence stretch participating in the binding. The sequence-affinity relationship is rather poorly understood. Thus, we usually cannot compare gene expression across different probes directly because signal intensity of the probe does not necessarily correlate with gene expression. This could be due to the cross-hybridization of the probe to a transcript of another gene, mapping of the probe to an intron, alternative splicing or single nucleotide polymorphism.

Affymetrix three-prime expression microarrays contain thousands of redundant probe sets that interrogate different regions of the same gene. For the cases where multiple probe sets represent the same gene, the assumption would be that the expression level changes should be consistent for all of those probe sets. Although this is a general assumption with microarray technology, it is not always the case, as demonstrated in **Figure 9**. Differential expression analysis methods rarely consider probe redundancy, which can lead to inaccurate inference about overall gene expression, or cause investigators to overlook potentially valuable information about differential regulation of variant mRNA products. Multiple probe sets representing the same gene poses a very practical issue for microarray data analysis and interpretation [28]–[30]. As demonstrated above (**Figure 10** and **Figure 11**), the inaccurate annotations for some probes can lead to a wrong conclusion in microarray data analysis. In order to correct the annotation issue, some research groups have developed computationally efficient tools to regroup the individual probes into consistent probe sets and then remap the probe sets to the correct sets of mRNA transcripts [31], [32]. However, it is difficult for third-party annotations to become widely adopted in place of the more commonly used Affymetrix annotations. Splice variants constitute an additional dimension of difficulty in microarray data analysis, as a single gene may have a large number of potential variants. A given short nucleotide probe targets either a constitutive exon (present in all splice variants) or an exon specific for certain splice variants. In the latter case, the specific splice variant will be measured, but other variants of the same gene will be ignored. Consequently, not all probe sets on Affymetrix arrays can represent entire genes, as shown in **Figure 12**.

RNA-Seq is a powerful technology that is predicted to replace microarrays for transcriptome profiling [9]. Compared to microarray, RNA-Seq avoids technical issues in microarray studies related to probe performance such as cross-hybridization, limited detection range of individual probes, as well as non-specific hybridization. Because it does not require probe design, it is devoid of the issues inherent with probe annotation. However, there are challenges involved with RNA-Seq that is currently limiting its potential utilization. The cost of RNA-Seq is more expensive than microarray, and thus RNA-Seq may be impractical for large studies. RNA-Seq is relatively new to most researchers, and the tools for RNA-Seq data analysis are far from mature. The lag between the development of data analysis tools and the speed with which RNA-Seq technology is advancing is already creating a data bottleneck for many users. Sequence reads in RNA-Seq are typically short, and do not always map uniquely to a single gene or isoform. Paralogous gene families, low-complexity sequence and high sequence similarity between alternatively spliced isoforms of the same gene are primary factors contributing to mapping uncertainty. As a consequence, a significant number of reads are multireads: reads that have high-scoring alignments to multiple positions in a reference genome or transcript set. How to assign multireads to genes remains a problem in reads mapping.

RNA-Seq analysis is vulnerable to the general biases and errors inherent in the next-generation sequencing (NGS) technology upon which it is based. The fragments are not uniformly sampled and sequenced, as there is variability in sequencing depth across the transcriptome due to preferential sites of fragmentation, variable primer and tag nucleotide composition effects [33], [34]. To address these biases, within- and between-sample correction and normalisation procedures have to be applied to correct sequence error, nucleotide composition, length or library preparation biases [35]–[38]. These approaches yield improvements in the corresponding RNA-Seq read counts with expression estimates gained by other experimental approaches. As sequencing technology advances, RNA-Seq experiments will continue generating larger volumes of data at lower cost, presenting increasing challenges for data management, storage, and analysis [39], [40]. The amount of data produced by an RNA-Seq experiment can be staggering - orders of magnitude greater than microarrays. In our study, for instance, a typical raw CEL data file generated from Affymetrix HT HG-U133+ PM array was 5 MB, whereas RNA-Seq sequence data in FASTQ format was roughly 23 GB. The raw data alone increases 4,600-fold per sample. Fortunately, in recent years, cloud computing [41] has emerged as a viable option to quickly and easily acquire computational resources for large-scale RNA-Seq data storage and analysis.

RNA-Seq can detect novel transcripts and isoforms, map exon/intron boundaries, discover sequence variations and reveal splice variants. For the study of differential gene expression, RNA-Seq does not suffer from hybridization-based limitations associated with microarray such as background noise and saturation, or with probe set issues such as incorrect annotation and isoform coverage. RNA-Seq is more sensitive in detecting genes with very low expression and more accurate in detecting expression of extremely abundant genes. RNA-Seq also has a wider dynamic range than microarray. With manufacturers predicting increased read lengths, reduced costs and faster sequencing relative to existing platforms, the future of RNA-Seq technology appears to be both promising and routinely affordable for most researchers. It is expected that once the barriers to widespread use of RNA-Seq are overcome—higher cost, high data-storage requirements, and the absence of a gold standard for analysis—this technique will become the predominant tool for transcriptome analysis.

### Data availability

The raw RNA-Seq data from this study has been deposited at the NCBI sequence read archive under the accession number SRP026389, while the raw microarray data is available at the NCBI Gene Expression Omnibus with the accession number GSE48978.

## Supporting Information

Figure S1 **The correlation of gene expression for biological replicates in microarray.**
(JPG)

Figure S2 **The correlation of gene expression for biological replicates in RNA-Seq.**
(JPG)

Table S1 **Summary of RNA-Seq read mapping.**
(XLSX)

Dataset S1 **Annotation for all probes sets in the HT HG-U133+ PM array.**
(XLSX)

Dataset S2 **18,306 genes common to both RefGene and Affymetrix HT HG-U133+ PM.**
(TXT)

Dataset S3 **Microarray data for all 12 samples after RMA normalization.**
(XLSX)

Dataset S4 **Differential expression analysis results in microarray.**
(XLSX)

Dataset S5 **Raw read counts and corresponding RPKM for genes in RNA-Seq.**
(XLSX)

Dataset S6 **Raw read counts and corresponding RPKM for genes in RNA-Seq.**
(XLSX)

Dataset S7 **Differential analysis results using RPKM for RNA-Seq.**
(CSV)
