# Supplementary figures and images for: Comparison of RNA-Seq and Microarray in Transcriptome Profiling of Activated T Cells
Source: PLoS One. 2014 Jan 16;9(1):e78644. doi: 10.1371/journal.pone.0078644 (PMC3894192; doi:10.1371/journal.pone.0078644)

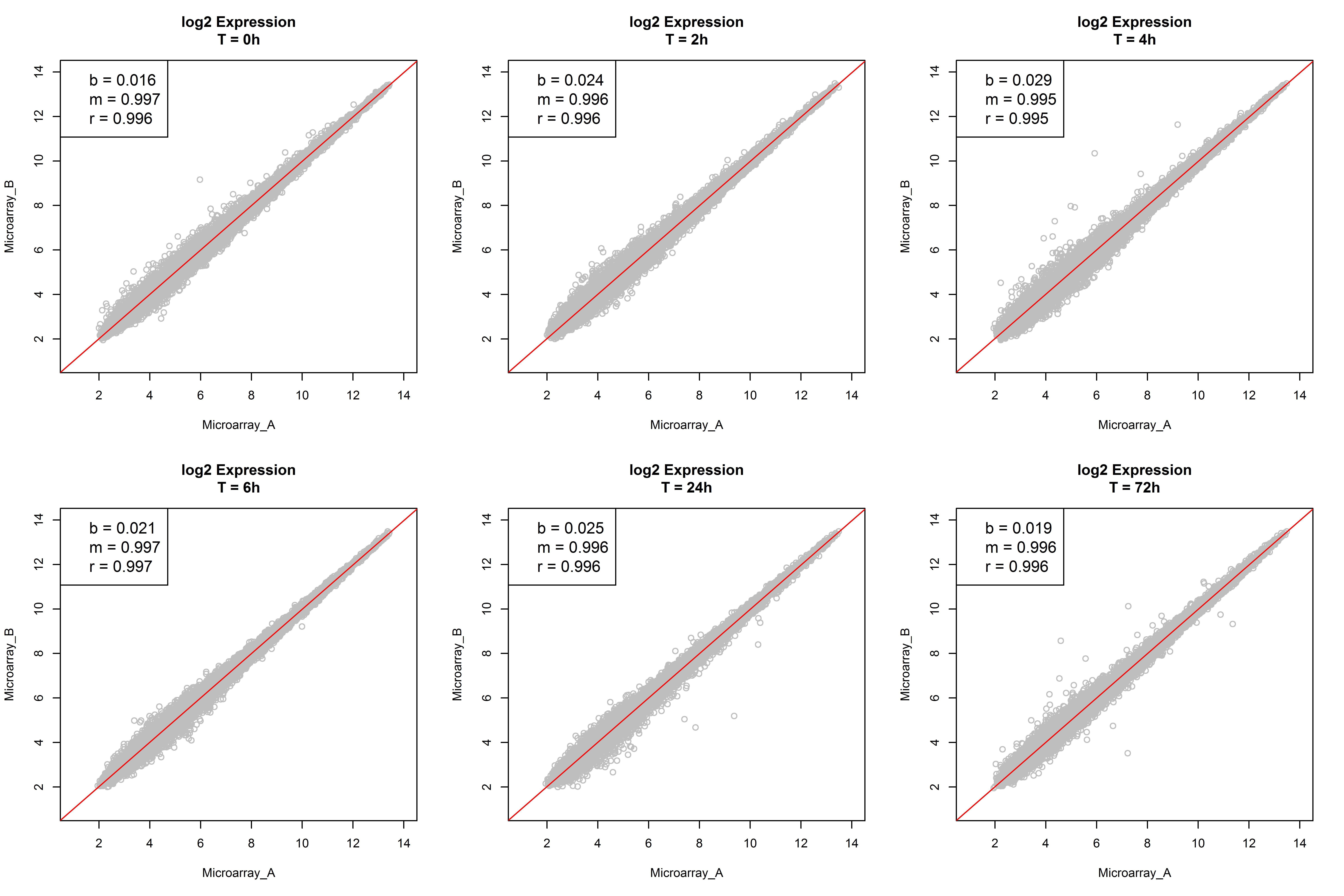

Supplement: Figure S1 — The correlation of gene expression for biological replicates in microarray. (JPG) [file pone.0078644.s001.jpg]

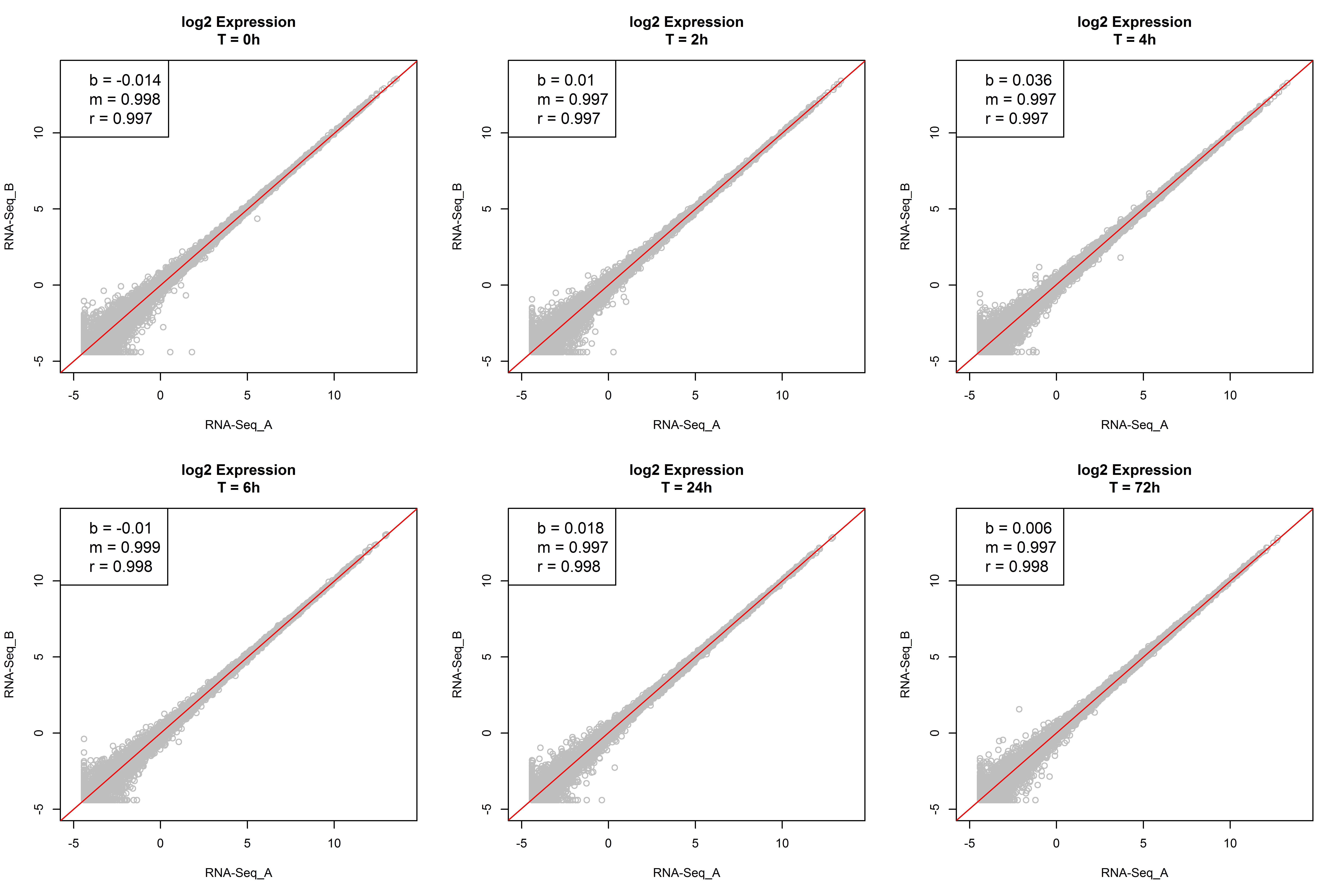

Supplement: Figure S2 — The correlation of gene expression for biological replicates in RNA-Seq. (JPG) [file pone.0078644.s002.jpg]
